# The Chemical Composition of Soyhulls and Their Effect on Amino Acid and Nutrient Digestibility in Laying Hens during the Peak of Production

**DOI:** 10.3390/ani13172808

**Published:** 2023-09-04

**Authors:** Muhammad Shuaib, Deependra Paneru, Abdul Hafeez, Muhammad Tahir, Woo Kyun Kim

**Affiliations:** 1 Department of Poultry Science, The University of Agriculture, Peshawar 25130, Pakistan; shoaibwzr@gmail.com (M.S.); hafeezpak@hotmail.com (A.H.); 2 Department of Poultry Science, University of Georgia, Athens, GA 30602, USA; dpaneru@uga.edu; 3 Department of Animal Nutrition, The University of Agriculture, Peshawar 25130, Pakistan; tahir065@yahoo.co.uk

**Keywords:** amino acids, digestibility, laying hen, peak production, soyhulls

## Abstract

**Simple Summary:**

Soyhulls, a by-product of soybean seed processing after oil extraction, have gained attention as a potential feed ingredient for poultry. This study evaluated the effects of 25% soyhull inclusion on nutrient and amino acid digestibility in laying hens at peak egg production. The hens were provided with either a corn–soybean meal diet or a soyhull diet. The soyhull diet had lower energy and digestibility of most nutrients and amino acids, except for nitrogen-free extract and methionine. However, the soyhull diet reduced uric acid excretion. This study suggests that soyhulls can partially replace soybean meal in laying hen diets.

**Abstract:**

This study investigates the chemical composition of soyhulls (SHs) as an alternative feed ingredient and their effect on nutrient and amino acid (AA) digestibility in laying hens during peak production. A total of 200 golden brown hens (28 weeks old) were subjected to random allocation across 5 dietary treatments: a corn–soybean meal (SBM) reference diet and 4 test diets with 25% SHs from different mills (SH1, SH2, SH3, and SH4). Each treatment was replicated four times with ten birds per replicate. Digesta samples were collected during three phases (28–32, 32–36, and 36–40 weeks of age) to measure apparent metabolizable energy (AME), the apparent ileal digestibility (AID) of nutrients, and the standard ileal digestibility (SID) of AAs. The SBM diet had 30.0% crude protein (CP) and 3.78% crude fiber (CF), while the SH diets had 21.0 to 21.5% CP and 11.6% CF. The findings revealed that the AME was lower (*p* < 0.05) with SH diets (2404 kcal/kg) compared to the SBM diet (2627 kcal/kg) in all three phases. The SH diets had a lower AID of dry matter (DM), crude protein (CP), ash, ether extract (EE), and crude fiber (CF) than the SBM diet by an average of 2.88, 2.25, 4.93, 4.99, and 3.36%, respectively. The AID of nitrogen-free extract (NFE) was higher in the SH diets than the SBM diet by 3.42% in all three phases (*p* < 0.05). The SH diets had lower uric acid excretion (about 66.93 mg/100 mL) than the SBM diet (about 76.43 mg/100 mL) on average in all three phases. The SH diets had a lower SID of arginine, histidine, isoleucine, lysine, cysteine, valine, and tyrosine than the SBM diet by 2 to 10%, while the SID of methionine was higher in the SH diets than the SBM diet by 2.2% on average in all three phases (*p* < 0.05). The SH from Sadiq Brother Feed (SH1) had the highest AME and AID of DM, ash, CP, EE, CF, and the SID of AA among the SH diets. These results indicate that SH can partially replace SBM in laying hen diets, but the source and quality of SH should be considered.

## 1. Introduction

The global landscape of animal agriculture is significantly shaped by the poultry production sector. The demand for poultry products, especially eggs, is increasing due to their high nutritional value, low cost, and wide acceptance by consumers. However, the cost of feed is a major challenge for poultry producers, as it accounts for 70 to 80% of the total production expenses [1]. Hence, the quest for substitute feed components that can lower feed expenses while enhancing feed efficiency holds crucial significance for ensuring the sustainability and economic viability of the poultry sector. Soybean meal (SBM) is extensively utilized as a protein source in poultry nutrition due to its elevated protein levels, well-balanced amino acid composition, and widespread availability [2]. SBM is also subject to price fluctuations and competition with human food and biofuel industries [3,4]. Furthermore, SBM contains anti-nutritional components like trypsin inhibitors, lectins, and phytates, which have the potential to hinder protein digestion and mineral assimilation in poultry [5]. Therefore, there is a need to explore other sources of protein and energy that can partially or completely replace SBM in poultry diets.

In this context, soyhulls (SHs) emerge as a promising alternative. SHs are derived from soybean seeds following the extraction of oil, and they hold significant value as a feed component for various animals, including ruminants and poultry [6,7]. With global soybean production projected to scale to an impressive 371.3 million tons by 2030, potentially yielding 29.7 to 37.1 million tons of soyhulls, their significance has become more pronounced [8]. Comprising approximately 9% crude protein (CP), 85.7% total carbohydrates, 1% lipids, and 4.3% ash on a dry matter (DM) basis, SHs offer a spectrum of advantages [9]. SHs as feed ingredients for poultry have several advantages: low-cost, readily available, easily stored and transported, high digestibility, and high metabolizable energy value due to their rapid fermentation and substantial pectin content [10]. Yet, SHs are not without limitations. They have a high fiber content that may reduce feed consumption and nutrient utilization and contain some anti-nutritional factors that may affect protein digestion and mineral absorption [11,12]. Dietary fiber (DF) is an essential component of poultry diets, as it affects various aspects of digestive physiology, nutrient utilization, gut health, and performance [13]. DF can be classified into soluble and insoluble fractions, depending on their solubility in water. Soluble fiber sources contain hygroscopic compounds (pectin, gum, and mucilage) that can increase the viscosity of the digesta, decrease the passage rate, and impair nutrient absorption [14]. Insoluble fiber sources contain structural polysaccharides (cellulose, hemicellulose, and lignin) that stimulate gizzard development, increase feed retention time, and enhance nutrient digestibility [15]. The optimal level and type of DF inclusion in poultry diets depend on a variety of factors, such as the source, form, method of processing, age, species, and production stage of the birds.

Prior research has indicated that adding 3 to 5% of DF to poultry diets does not adversely affect nutrient digestibility or growth performance [16,17,18,19]. However, the results from DF studies are inconsistent, indicating the need for further investigation on the effects of DF inclusion level and type on poultry nutrition [19,20]. SHs are a potential source of DF that have been tested in many animals, but their effects on laying hens have not been well-studied, especially during the peak production period. The peak production period is critical for laying hens, as it determines their egg production potential and profitability. Therefore, it is important to evaluate the impact of SH inclusion on nutrients and amino acid (AA) digestibility during this stage. Thus, the aim of this study was to evaluate the chemical composition of SHs from different sources and their effect on AME, the standard ileal digestibility (SID) of AA, fecal uric acid content, and proximate analysis values in golden brown (RIR × Fayoumi) laying hens during the peak egg production period.

## 2. Materials and Methods

This research received approval from the Ethics Committee at the University of Agriculture Peshawar, Pakistan (protocol No. 6780-A/LM, B&G/UOA; Approval Date: 31 December 2020). The handling of the birds adhered to the established guidelines for animal care and utilization outlined in the “Guide for the Care and Use of Agricultural Animals in Agricultural Research and Teaching” [21].

### 2.1. Birds and the Experimental Diets

A total of 200 golden brown laying hens, which are a cross between Rhode Island Red and Fayoumi breeds and have an average body weight of 1.4 kg and at 28 weeks of age and peak production were randomly allocated to 100 cages, with two birds per cage. The cage was equipped with an individual feeder, a nipple-based drinker, and a feces collection tray. The birds were vaccinated according to a standard schedule and reared in a controlled environment with an average daily temperature of 24 °C.

The birds were randomly allocated to 5 dietary treatments with 4 replicates of 10 birds each. The basal diet was supplemented with 1% Celite (Celite Corp., Lompoc, CA, USA), an indigestible marker to determine the ileal nutrient digestibility. Test ingredients (soyhulls) from four different feed mills (Sadiq Brother Feed, Mandra, Shabbir Feed, Multan, Sindh Feeds, Karachi, and Hi-Tech Feeds, Lahore) across Pakistan were used in this study. A basal diet was formulated using corn–soybean meal to meet or surpass the nutrient requirements of laying hens. The reference diet (SBM) contained 25% soybean meal (as is) and 75% basal diet (as is). Test diets (SH1, SH2, SH3, and SH4) contained 25% soyhulls (as is) from each feed mill and 75% basal diet (as is), respectively. All diets received equal supplementation of vitamins, minerals, and additional non-energy ingredients. The ingredient composition of the nitrogen-free diet (NFD), basal, and experimental diets (fed as mash) are presented in Table 1. Experimental diets were fed to the birds from 28 to 40 weeks of age in three phases (phase 1: 28 to 32 weeks, phase 2: 32 to 36 weeks, and phase 3: 36 to 40 weeks). Feed intake was recorded, and feces samples were collected and analyzed during the last three days of each phase.

### 2.2. Chemical Analysis

Test ingredients (i.e., soybean meal and four different soyhulls), experimental diets, and ileal digesta were analyzed for proximate composition: DM, moisture, CP, EE, CF, and ash, according to the methods of [22]. An adiabatic bomb calorimeter (AC500, Leco, St. Joseph, MI, USA) standardized with benzoic acid was used to determine the gross energy of experimental diets and the ileal digesta. The AA profile of the test ingredients was quantified in an AA analyzer (Biochrom 30+, Biochrom Ltd., Cambridge, UK). The test ingredients were ground to pass out a 0.5 mm screen. The samples were then hydrolyzed with 6N HCl containing 0.1% (*w*/*v*) phenol for 24 h at 110 ± 2 °C in glass tubes sealed under vacuum. The AAs were then separated by ion exchange chromatography, and their absorbance was measured simultaneously at 570 and 440 nm. Cysteine and methionine were measured as cysteic acid and methionine sulfone through a process involving oxidation with performic acid over a 16 h period at a temperature of 0 °C. Subsequently, neutralization was carried out using hydrobromic acid before proceeding to hydrolysis.

### 2.3. Ileal AA Digestibility Assay

Two birds from each replicate were euthanized by cervical dislocation at the end of each phase, and ileal digesta were collected from the lower half of the ileum, specifically from Meckel’s diverticulum to a point about 40 mm proximal to the ileocecal junction. The ileal digesta from two birds were pooled within a replicate and collected into a plastic bag. The pooled ileal digesta samples were freeze-dried and stored at −20 °C until the analysis. The experimental diets and freeze-dried samples of ileal digesta were finely ground to pass through a 0.5 mm sieve. Subsequently, they were stored in sealed plastic bags under refrigeration at 4 °C until analysis. Experimental diets and ileal digesta were analyzed for AA composition using an AA analyzer (Biochrom 30+, Biochrom Ltd., Cambridge, UK).

### 2.4. Calculations

Apparent metabolizable energy (AME) values (kcal/kg of DM) of the experimental diets were calculated using the following equation [23]:

AME = GE_Diet_ − [GE_Excreta_ − (AIA_I_/AIA_O_)] (1)

where AME is the apparent metabolizable energy (kcal/kg DM of diet), GE_Diet_ is the gross energy of feed (kcal/g DM), and GE_Excreta_ is the gross energy of excreta (kcal/g DM). AIA_I_ is the acid-insoluble ash concentration of dietary intake and AIA_O_ is the acid-insoluble ash concentration of ileal output.

Apparent ileal digestibility (AID) values were calculated using the index method according to the following equation [24]:

AID_X_, % = 100 − [(AIA_I_/AIA_O_) × (N_O_/N_I_) × 100] (2)

where AID_X_ is the apparent ileal digestibility of DM, ash, CP, EE, CF, or NFE; AIA_I_ is the acid-insoluble ash concentration of dietary intake; AIA_O_ is the acid-insoluble ash concentration of ileal output; N_O_ is the nutrient concentration of ileal output; and N_I_ is the nutrient concentration of dietary intake. All values for AIA_I_, AIA_O_, N_O_, and N_I_ are expressed as mg/kg of DM.

Endogenous losses of amino acids (EALs) were calculated using the following equation [25]:

EAL = AAd × (AIA_IN_/AIA_ON_) (3)

where the EAL is the endogenous loss of an amino acid (mg/kg DM intake), AAd is the concentration of that amino acid in digesta (mg/kg DM), AIA_IN_ is the acid-insoluble ash concentration of the N-free diet, and AIA_ON_ is the acid-insoluble ash concentration of ileal output from birds fed the N-free diet.

Standardized ileal digestibility (SID) of AAs was calculated by adding values for apparent ileal digestibility (AID) and the endogenous loss of an amino acid (EAL) according to the following equation [26]:

SID = AID + (EAL/AAf) (4)

where SID signifies the percentage value of the standardized ileal digestibility coefficient for amino acids. AID denotes the percentage value of the apparent ileal digestibility coefficient, which is calculated using Equation (2), EAL represents the measured non-specific endogenous loss of the specific amino acid at the distal ileum (mg/kg DMI) after feeding the N-free diet, and is calculated according to Equation (3), and AAf corresponds to the amino acid content within the diet, measured in mg/kg of dry matter.

Uric acid content (UA) of the excreta was determined by a simple spectrophotometric technique using the following equation [27]:

UA = (A × 100 × 168.1 × 15)/∑(5)

where UA is the uric acid content (mg/g feces), A is the absorbance of the sample at 285 nm for a 1 cm light path, 100 is the volume of the original extraction solution in mL, 168.1 is the molecular weight of uric acid, 15 is the dilution factor, and ∑ is the molar extinction coefficient at a given wavelength (i.e., 11,500 at 285 nm).

### 2.5. Statistical Analysis

Data collected were analyzed by the GLM procedure in SPSS 21.0 (IBM Corp, Armonk, NY, USA) using a one-way ANOVA model with diet as the main factor. Replicate pens were considered experimental units. Statistical significance was declared at *p* < 0.05, and a *p*-value between 0.05 and 0.10 was considered a trend. Post hoc comparisons of means were performed using a Tukey test.

## 3. Results

### 3.1. Nutrient Profile of the Test Ingredients

The nutritional composition of the ingredients used to formulate experimental diets is presented in Table 2. SBM had higher levels of CP (46.3%), ash (6.98%), and total AA (46.23%) compared to SHs from various sources. On the other hand, SHs had higher levels of EE (ranging from 1.9 to 2.2%) and CF (ranging from 31.5 to 33.8%) compared to SBM. SBM contained 20.75% essential AAs, whereas SHs ranged from 4.07 to 4.19%. Regarding non-essential AAs, SBM contained 25.48%, while SHs ranged from 5.16 to 5.29%. Leucine was the most abundant essential AA in SBM (3.56%), while lysine was the most abundant in SHs (ranging from 0.72 to 0.74%). Methionine was the least abundant essential AA in both SBM (0.6%) and SHs (0.13%). Among the non-essential amino acids, glutamic acid stood out as most abundant in both SBM (8.72%) and SHs (ranging from 1.20 to 1.24%), while cysteine was the least abundant in both SBM (0.67%) and SHs (ranging from 0.17 to 0.18%). SHs from different sources showed similarities in terms of DM, EE, CF, and total AAs. Among the different sources of SHs, SH1 had the highest CP content (12.5%), while SH3 had the lowest (11.4%). The analyzed AA values of the test ingredients were employed to determine the AA levels within the experimental diets.

### 3.2. Apparent Ileal Digestibility of Nutrients

The higher apparent metabolizable energy (AME) was observed in the soybean meal (SBM) diet (2627 kcal/kg) compared to the soyhull (SH) diets (2404 kcal/kg) (Table 3). Among the SH diets, SH1 consistently had the highest AME values. The SH diets resulted in an average decrease of 2.88% in apparent ileal digestibility (AID) of dry matter (DM) compared to the SBM diet. There were no notable differences in DM digestibility within the SH diets, except in phase 3, where SH3 had the lowest value.

Furthermore, the SH diets contributed to an average reduction of 4.93% in the AID of ash relative to the SBM diet. The AID of crude protein (CP) demonstrated an average decrease of 2.25% in the presence of the SH diets in contrast to the SBM diet. Notably, during phase 2, the SBM diet exhibited comparable CP digestibility to the SH1 and SH2 diets, while it was higher than the digestibility observed in the SH3 and SH4 diets. Additionally, the SH diets displayed an average decrease of 4.99% in the digestibility of ether extract (EE) compared to the SBM diet. Among the SH diets, SH1 presented the highest EE digestibility, whereas SH3 displayed the lowest values during phases 1 and 2. No significant differences were observed among the SH diets in phase 3.

In terms of crude fiber (CF) digestibility, the SBM diet demonstrated a higher value by an average of 3.36% compared to the SH diets. Specifically, in phase 2, CF digestibility was lower in the SH3 and SH4 diets relative to the SH1 and SH2 diets. The nitrogen-free extract (NFE) digestibility was lower in the SBM diet by an average of 3.42% compared to the SH diets. During phase 1, the SH1, SH2, and SH4 diets exhibited higher NFE digestibility than the SH3 diet.

Overall, the SH diets exhibited a lower AME and AID of nutrients in comparison to the SBM diet, with notable variations observed among different SH diets and across the phases.

Fecal uric acid (FUA) excretion was consistently higher in the SBM diet (about 76.43 mg/100 mL) compared to the SH diets (about 66.93 mg/100 mL). Furthermore, among the SH diets, SH2 displayed the highest FUA during phases 1 and 2, whereas SH1 exhibited the lowest. In phase 3, SH2 demonstrated the highest value, followed by SH4, SH3, and SH1. The specific FUA content (mg/100 mL) of SBM and SH1, SH2, SH3, and SH4 diets were 76.4, 67.1, 68.4, 67.8, and 67.4 in phase 1; 74.9, 63.0, 66.0, 63.8 and 65.5 in phase 2; and 78.0, 65.9, 71.5, 67.9 and 68.9 in phase 3, respectively.

### 3.3. Standardized Ileal AA Digestibility

The SID coefficient AAs for the SBM and SH diets for three phases (phase 1: 28 to 32 weeks, phase 2: 32 to 36 weeks, and phase 3: 36 to 40 weeks of age) are presented in Table 4, Table 5 and Table 6**,** respectively. The SH diets reduced the SID of arginine, histidine, isoleucine, lysine, cysteine, valine, and tyrosine more than the SBM diet by approximately 4.6, 5.5, 5.8, 3.9, 2.3, 9.1, and 9.7%, respectively, while the SID of methionine increased in the SH diets more than the SBM diet by 2.2% on average in all three phases (*p* < 0.05).

During phase 1, the SBM diet had higher SID (*p* < 0.05) for most essential AAs and non-essential AAs than the SH diets. The exceptions were leucine, methionine, and tryptophan for essential AAs and proline for non-essential AAs, which were unaffected by the experimental diets (*p* > 0.05). The SID of alanine was higher for the SH diets than the SBM diet, except for SH2, which had similar values to the SBM diet. The total essential AAs, non-essential AAs, and total AA digestibility were lower in the SH diets than the SBM diet.

Similarly, during phase 2, the SBM diet had a higher SID (*p* < 0.05) for most essential AAs and non-essential AAs than the SH diets. The exceptions were methionine for essential AAs and alanine, glycine, and proline for non-essential AAs, which were unaffected by the experimental diets (*p* > 0.05). The total essential AAs, non-essential AAs, and total AA digestibility were lower in the SH diets than the SBM diet.

During phase 3, the SBM diet had higher SID coefficients (*p* < 0.05) for most essential AAs and non-essential AAs than the SH diets (Table 6). The exceptions were leucine for essential AAs and alanine and proline for non-essential AAs, which were unaffected by the experimental diets (*p* > 0.05). The SID coefficient of methionine was higher in the SH diets than the SBM diet (*p* < 0.05). The total essential AAs, non-essential AAs, and total AA digestibility were lower in the SH diets than the SBM diet (*p* < 0.05). Among the SH diets, SH1 had a numerically higher SID for most of the AAs than SH2, SH3, and SH4 in all three phases.

## 4. Discussion

The purpose of this study was to compare the nutrient and AA digestibility in laying hens fed diets containing either 25% of soyhulls (SHs) from different origins or 25% of soybean meal (SBM) as a replacement for basal diet during the peak egg production period (28 to 40 weeks of age). It is crucial to consider that laying hens have increased nutrient requirements during the peaking period to compensate for the energy and nutrient requirements for egg production [28]. As laying hens undergo peak production, their dietary needs become pivotal in sustaining optimal egg production. This phase is characterized by increased nutrient requirements to overcome the increased energy expenditure associated with egg production. The inclusion of SHs in the corn–soybean meal diet introduces both soluble and insoluble fiber components, potentially causing shifts in nutrient composition and overall dietary dynamics [29]. This alteration arises from the dilution of digestible nutrients, resulting in a feed with lower energy and increased bulkiness. Numerous studies have reported the increased excretion and decreased digestibility of nutrients when higher numbers of soyhulls are included in the diet, resulting from the elevated dietary fiber in the diet [7,30,31].

In the current study, the decrease in the AME of the SH diets resulted from the direct replacement of high-energy SBM with fibrous soyhulls, which may be due to reduced digestibility of proteins, carbohydrates, and lipids in the diet by forming a physical barrier that might have limited the interaction between digestive enzymes and nutrients in the gastrointestinal tract of laying hens [32]. This disparity underscores the inherent differences in energy density between the two feed ingredients. It is widely recognized that fibrous feedstuff has a negative impact on nutrient and energy retention in poultry, which can be partly explained by the inhibitory effects of fiber on digestive processes [11]. Our results were consistent with the study by Leung et al. [12], who reported a decrease in AME when SHs were included at high levels (40%) in the diet of broiler breeder hens. However, within the SH sources, the SH1 diet had higher AME than others, indicating potential variability in energy availability across different sources of soyhulls. This suggests that the quality and variability of SHs may affect their energy value for laying hens. The differences in nutrient composition among SH sources could be related to the processing conditions, such as temperature and moisture, and the degree of hull removal from the soybean seeds [33].

The ileal digestibility of dry matter (DM) was, on average, 2.5% lower for the SH diets than the SBM diet. SHs contain a higher proportion of dietary fiber, particularly insoluble fiber, than soybean meal [34]. Insoluble fibers, such as cellulose and lignin in SHs, are resistant to enzymatic breakdown by endogenous enzymes in the intestine [35]. Insoluble fibers pass through the digestive tract relatively undigested and contribute to the fecal bulk. This reduces the overall digestibility of DM in diets containing higher levels of SHs. Similar results were observed in the growing pigs with the inclusion of SHs in the SBM-based diet; the AID of DM was linearly decreased with the inclusion of 0, 3, 6, and 9% SHs [36]. The high fiber content in soyhulls can also impact nutrient availability and absorption. The presence of fiber can reduce the solubility and diffusion rate of nutrients in the intestinal lumen, limiting their accessibility to digestive enzymes and reducing their digestibility [32].

In the present study, the ileal digestibility of CP was, on average, 2.25% lower in the SH diets than in the SBM diet. It might be due to lower CP content and higher fiber content in SHs than SBM, which can reduce the amount and availability of protein for digestion and absorption in the intestine. Moreover, the high fiber content can also interfere with the activity and availability of digestive enzymes by binding to them or diluting their concentration, as observed in some in vitro and human studies [35,37,38]. Furthermore, the high fiber content in soyhulls can alter the gut environment, such as pH and microbial populations, which can influence the breakdown and absorption of dietary protein by gut microorganisms [39]. These factors can result in lower enzymatic breakdown and subsequent lower digestibility of CP in the intestine.

The ileal digestibility of CF was, on average, 3.4% lower in the SH diets than the SBM diet. SHs have a higher CF content than SBM, and are mainly composed of insoluble fiber components, such as cellulose and lignin. These fiber components have complex structures resistant to enzymatic breakdown in the intestine. Laying hens have limited endogenous enzymes capable of efficiently breaking down complex carbohydrates and fiber, such as cellulases and hemicellulases [40]. Moreover, the enzymes produced by the hens may have lower efficacy in digesting the specific types of fiber present in soyhulls. As a result, the CF present in soyhulls is less accessible to the digestive enzymes, leading to reduced digestibility.

Uric acid is the end product of purine metabolism in birds, and its excretion is influenced by dietary factors, such as protein and fiber content [41]. In the present study, the excreted uric acid content was lower in the SH diets (~67.5 mg/100 mL) than the SBM diet (~76 mg/100 mL). SBM has a higher CP content than SHs (47.5% vs. 9.7% on a dry matter basis), which means it contains more purines than SHs [41]. Therefore, feeding SBM may result in higher uric acid production and excretion than feeding SHs. The difference can also be attributed to the high fiber content, particularly insoluble fiber, present in SHs. The high fiber content in SHs allows uric acid and other waste products to bind to the digestive tract [42]. This binding action reduces the solubility and availability of uric acid for excretion, resulting in decreased excretion of uric acid in the feces of laying hens. It is worth noting that similar findings have been reported in other studies. Such et al. [43] observed decreased total nitrogen and uric acid contents in the excreta of broilers fed a low protein diet, and Namroud et al. [44] also reported decreased uric acid, moisture, and acidity of excreta in broilers with a reduced amount of dietary crude protein. Additionally, Roberts et al. [45] observed lower uric acid content in the feces of laying hens when fed fiber sources such as soyhulls, wheat middling, or distiller dried grains with solubles. One of the reasons for the reduced uric acid excretion in feces is that apart from providing energy, fiber also serves as a source of nitrogen for bacteria. The bacteria utilize nitrogen from the fiber, which would otherwise be excreted as uric acid, shifting nitrogen excretion from uric acid to bacterial protein [13]. These findings align with previous studies that have shown a decrease in ammonia (NH_3_) emissions and nitrogen excretion in pigs, broilers, and laying hens when high-fiber diets with reduced crude protein content are fed [46,47,48].

Consistent with previous findings, the SH diets reduced the SID of most of the AAs, including arginine, histidine, isoleucine, lysine, cysteine, valine, and tyrosine [49]. The reduction in the SID of AAs observed with the inclusion of high levels of SHs in the diet may be attributed, at least in part, to the higher lignin content present in SHs compared to SBM [31]. It is well-established that the digestibility of nutrients in feedstuffs is inversely correlated with the degree of lignification exhibited by those feedstuffs [50]. SHs also have a higher fiber content than SBM, especially insoluble fiber, which can interfere with the activity and availability of digestive enzymes by binding to them or diluting their concentration. Furthermore, the differences between insoluble and soluble fibers could explain the observed differences in the SID of AAs. Insoluble dietary fiber (IDF) is more abrasive than soluble dietary fiber (SDF), and as a result, IDF scrapes more mucin from the intestinal mucosa during its passage through the gastrointestinal tract [31]. The presence of an anti-nutritional factor, phytate, can also limit the AA digestibility in the SH diets [51]. Phytates can bind to dietary protein electrostatically and form protein–phytate complexes [52]. These complexes are resistant to enzymatic protein digestion and can reduce the digestibility of amino acids in the ileum [53]. The results of the present study agree with previous studies that reported lower amino acid digestibility in poultry-fed high-fiber diets containing SHs or other fiber sources [49,54]. However, other studies have reported higher or similar amino acid digestibility in poultry-fed diets containing SHs or extruded SHs [55]. These discrepancies may be due to differences in animal species, age, feed processing, fiber source, level of inclusion, and experimental methods. The present study also showed that among the test diets, SH1 had numerically higher SID coefficients for most amino acids than SH2, SH3, and SH4 in all three phases. This may be attributed to the differences in the origin and quality of SHs from different sources [7].

## 5. Conclusions

In conclusion, the inclusion of 25% soyhulls in the corn–soybean meal-based diet of golden brown (RIR × Fayoumi) laying hens did not produce adverse effects on their apparent metabolizable energy (AME) and nutrient and amino acid digestibility. However, it was observed that soyhull supplementation led to a reduction in fecal uric acid levels. Among the different soyhulls obtained from various feed mills, those from Sadiq Brother feed mills (SH1) performed better when included at a level of 25% in the basal diet. These results indicate that soyhulls can partially replace soybean meal in laying hen diets, but the source and quality of soyhulls should be considered.

## Figures and Tables

**Table 1:** Ingredient and analyzed composition of the experimental diets.

Ingredient, %	NFD ^4^	Basal Diet	Experimental Diets ^5^				
(Fed Basis)			SBM	SH1	SH2	SH3	SH4
Corn Starch	17.0	0	0	0	0	0	0
Dextrose	64.0	0	0	0	0	0	0
Arbocel ^1^	5.0	0	0	0	0	0	0
Corn	0	58.0	43.5	43.5	43.5	43.5	43.5
Soybean Meal	0	35.0	51.2	26.2	26.2	26.2	26.2
Soyhulls	0	0	0	25.0	25.0	25.0	25.0
Soybean Oil	5.0	2.00	1.50	1.50	1.50	1.50	1.50
Dicalcium Phosphate	2.0	2.10	1.58	1.58	1.58	1.58	1.58
Limestone	1.5	0.80	0.60	0.60	0.60	0.60	0.60
Sodium Bicarbonate	1.5	0.35	0.26	0.26	0.26	0.26	0.26
Choline Chloride	0.4	0	0	0	0	0	0
Sodium Chloride	0.2	0.25	0.19	0.19	0.19	0.19	0.19
Potassium Chloride	1.6	0	0	0	0	0	0
Premix ^2^	0.80	0.50	0.37	0.37	0.37	0.37	0.37
Celite ^3^	1.0	1.00	0.75	0.75	0.75	0.75	0.75
Analyzed Values							
Dry Matter	-	-	88.5	87.4	87.3	87.3	87.3
Ash (DM Basis)	-	-	5.89	4.87	4.74	4.81	4.78
CF (DM Basis)	-	-	3.78	11.6	11.6	11.6	11.6
EE (Feed Basis)	-	-	5.46	3.68	3.62	3.65	3.64
EE (DM Basis)	-	-	6.16	4.21	4.14	4.17	4.16
CP (DM Basis)	-	-	30.0	21.5	21.0	21.4	21.2
NFE (Fed Basis)	-	-	53.1	57.1	57.3	57.3	57.3
Acid Insoluble Ash	-	-	1.37	1.08	1.05	1.07	1.06

^1^ ARBOCEL^®^ powdered cellulose is a plant-based functional filler (JRS Pharma, Rosenberg, Germany). ^2^ Premix provided per kg of diet: vitamin A, 4400 IU; vitamin E, 12 IU; vitamin D_3_, 118 µg; thiamine, 2.5 mg; menadione sodium bisulfate, 2.40 mg; niacin, 30 mg; vitamin B_2_, 4.8 mg; D-pantothenic acid, 10 mg; vitamin B_6_, 5 mg; vitamin B_7_, 130 µg; cyanocobalamin, 19 µg; vitamin B_9_, 2.5 mg; manganese, 85 mg; Zinc, 75 mg; iron, 80 mg; iodine, 1 mg; selenium, 130 µg; copper, 6 mg. ^3^ Celite: a source of acid-insoluble ash used as an indigestible biomarker for digestibility (Celite Corp., Lompoc, CA, USA). ^4^ NFD: N-free diet formulated to determine the basal endogenous AA losses. ^5^ Experimental diets: SBM = reference diet prepared by substituting 25% of the basal diet with soybean meal; SH1 = test diet prepared by substituting 25% of the basal diet with soyhulls sourced from Sadiq Brother Feed, Mandra, Pakistan; SH2 = test diet prepared by substituting 25% of the basal diet with soyhulls sourced from Shabbir Feed, Multan, Pakistan; SH3 = test diet prepared by substituting 25% of the basal diet with soyhulls sourced from Sindh Feeds, Karachi, Pakistan; SH4 = test diet prepared by substituting 25% of the basal diet with soyhulls sourced from Hi-Tech Feeds, Lahore, Pakistan.

**Table 2:** Analyzed composition of ingredients on an as-fed basis.

Item, %	Test Ingredients ^1^				
	SBM	SH1	SH2	SH3	SH4
Dry Matter	92	88.9	88	88.5	87.9
Moisture	8	11.1	12	11.5	12
Crude Protein	46.3	12.5	11.8	11.4	12.3
Ether Extract	0.85	2.2	2.2	2	1.9
Crude Fiber	4.37	31.5	33.4	32.2	33.8
Ash	6.98	4	4.2	4.5	4.4
Essential AAs					
Arginine	3.39	0.54	0.52	0.54	0.53
Histidine	1.29	0.28	0.28	0.26	0.27
Isoleucine	2.08	0.41	0.41	0.4	0.39
Leucine	3.56	0.7	0.68	0.69	0.68
Lysine	2.85	0.74	0.74	0.73	0.72
Methionine	0.6	0.13	0.13	0.13	0.13
Phenylalanine	2.38	0.41	0.4	0.39	0.4
Threonine	1.84	0.39	0.38	0.38	0.39
Tryptophan	0.62	0.11	0.1	0.11	0.1
Valine	2.14	0.48	0.47	0.46	0.46
Total EAA	20.75	4.19	4.11	4.09	4.07
Non-essential AAs					
Alanine	2.04	0.47	0.46	0.47	0.46
Aspartic Acid	5.35	1.02	1.01	1	0.99
Cysteine	0.67	0.18	0.18	0.17	0.17
Glutamic Acid	8.72	1.24	1.22	1.22	1.2
Glycine	2.35	0.88	0.89	0.88	0.87
Proline	2.32	0.56	0.55	0.55	0.54
Serine	2.39	0.58	0.56	0.56	0.57
Tyrosine	1.64	0.36	0.37	0.35	0.36
Total NEAA	25.48	5.29	5.24	5.20	5.16
Total AA	46.23	9.48	9.35	9.29	9.23

^1^ Test ingredients: SBM = soybean meal; SH1 = soyhulls from Sadiq Brother Feed, Mandra, Pakistan; SH2 = soyhulls from Shabbir Feed, Multan, Pakistan; SH3 = soyhulls from Sindh Feeds, Karachi, Pakistan; SH4 = soyhulls from Hi-Tech Feeds, Lahore, Pakistan.

**Table 3:** Apparent metabolizable energy (kcal/kg) and apparent ileal digestibility (%) of dry matter, ash, crude protein, ether extract, crude fiber, nitrogen-free extract, and uric acid excretion (mg/100 mL) of laying hens during peak egg production periods at different phases (phase 1: 28 to 32 weeks; phase 2: 32 to 36 weeks, and phase 3: 36 to 40 weeks).

Item	Experimental Diets ^1^					SEM	*p*-Value
	SBM	SH1	SH2	SH3	SH4		
Phase 1 (28 to 32 weeks)							
AME (kcal/kg)	2633 ^a^	2414 ^b^	2396 ^d^	2405 ^c^	2389 ^e^	5.54	0.003
Dry Matter, %	68.2 ^a^	66.2 ^b^	65.4 ^b^	64.2 ^b^	64.3 ^b^	0.51	0.002
Ash, %	54.0 ^a^	47.4 ^b^	47.2 ^b^	47.0 ^b^	46.3 ^b^	0.3	0.021
Crude Protein, %	79.0 ^a^	77.4 ^b^	77.0 ^b^	76.0 ^b^	76.3 ^b^	0.42	0.048
Ether Extract %	58.2 ^a^	56.2 ^b^	54.4 ^c^	55.4 ^bc^	55.0 ^bc^	0.26	0.035
Crude Fiber, %	80.4 ^a^	76.4 ^b^	75.3 ^b^	76.0 ^b^	76.2 ^b^	0.67	0.029
N-free Extract, %	70.2 ^b^	74.0 ^a^	73.2 ^a^	72.4 ^ab^	73.0 ^a^	0.37	0.03
Uric Acid (mg/100 mL)	76.4 ^a^	67.1 ^b^	68.4 ^b^	67.8 ^b^	67.4 ^b^	1.41	0.024
Phase 2 (32 to 36 weeks)							
AME (kcal/kg)	2632 ^a^	2434 ^b^	2430 ^b^	2365 ^d^	2412 ^c^	10.2	0.032
Dry Matter, %	69.4 ^a^	67.4 ^b^	66.2 ^b^	66.0 ^b^	66.0 ^b^	0.62	0.022
Ash, %	48.4 ^a^	45.5 ^b^	45.2 ^b^	44.0 ^b^	44.4 ^b^	0.27	0.013
Crude Protein, %	77.0 ^a^	76.2 ^ab^	76.0 ^ab^	75.4 ^b^	75.0 ^b^	0.38	0.03
Ether Extract %	57.0 ^a^	52.4 ^b^	52.0 ^b^	50.0 ^c^	51.0 ^bc^	0.3	0.016
Crude Fiber, %	79.0 ^a^	77.2 ^b^	76.0 ^bc^	75.4 ^c^	75.0 ^c^	0.73	0.038
N-free Extract, %	66.0 ^b^	70.2 ^a^	70.0 ^a^	69.0 ^a^	69.2 ^a^	0.23	0.031
Uric Acid (mg/100 mL)	74.9 ^a^	63.0 ^b^	66.0 ^b^	63.8 ^b^	65.5 ^b^	0.37	0.031
Phase 3 (36 to 40 weeks)							
AME (kcal/kg)	2617 ^a^	2438 ^b^	2406 ^c^	2377 ^e^	2387 ^d^	10.4	0.042
Dry Matter, %	68.0 ^a^	66.4 ^b^	65.4 ^bc^	64.4 ^c^	66.0 ^b^	0.32	0.033
Ash, %	51.4 ^a^	48.4 ^b^	48.0 ^b^	47.4 ^b^	47.2 ^b^	0.46	0.012
Crude Protein, %	80.4 ^a^	78.2 ^b^	76.4 ^bc^	77.3 ^c^	77.4 ^c^	0.38	0.006
Ether Extract %	60.2 ^a^	54.4 ^b^	54.2 ^b^	53.4 ^b^	53.3 ^b^	0.37	0.023
Crude Fiber, %	80.3 ^a^	78.4 ^b^	78.0 ^b^	77.4 ^b^	77.2 ^b^	0.46	0.041
N-free Extract, %	68.0 ^b^	72.2 ^a^	72.0 ^a^	71.4 ^a^	71.2 ^a^	0.27	0.028
Uric Acid (mg/100 mL)	78.0 ^a^	65.9 ^d^	71.5 ^b^	67.9 ^c^	68.9 ^c^	0.41	0.013

^a–e^ Means within the same row with different superscripts are significantly different at *p* < 0.05. ^1^ Experimental diets: SBM = reference diet prepared by substituting 25% of the basal diet with soybean meal; SH1 = test diet prepared by substituting 25% of the basal diet with soyhulls sourced from Sadiq Brother Feed, Mandra, Pakistan; SH2 = test diet prepared by substituting 25% of the basal diet with soyhulls sourced from Shabbir Feed, Multan, Pakistan; SH3 = test diet prepared by substituting 25% of the basal diet with soyhulls sourced from Sindh Feeds, Karachi, Pakistan; SH4 = test diet prepared by substituting 25% of the basal diet with soyhulls sourced from Hi-Tech Feeds, Lahore, Pakistan.

**Table 4:** Standardized ileal digestibility (%) of amino acids in laying hens fed experimental diets during phase 1 (28 to 32 weeks of age).

Item	Experimental Diets ^1^					SEM	*p*-Value
	SBM	SH1	SH2	SH3	SH4		
Essential amino acids							
Arginine	94.5 ^a^	91.0 ^b^	88.4 ^c^	88.8 ^c^	90.4 ^bc^	0.42	0.012
Histidine	92.9 ^a^	87.6 ^b^	87.4 ^b^	87.5 ^b^	87.2 ^b^	0.21	0.024
Isoleucine	90.3 ^a^	85.0 ^b^	84.4 ^bc^	83.8 ^bc^	85.0 ^b^	0.29	0.001
Leucine	85.6	86.5	86	85.7	86.2	0.23	0.051
Lysine	92.0 ^a^	89.2 ^b^	88.5 ^b^	88.0 ^b^	88.2 ^b^	0.18	0.003
Methionine	92.1	93.6	93.2	93	93.4	1.10	0.879
Phenylalanine	93.1 ^a^	88.5 ^b^	87.4 ^b^	88.2 ^b^	87.5 ^b^	0.15	0.013
Threonine	88.5 ^a^	85.5 ^b^	85.0 ^b^	84.8 ^b^	85.2 ^b^	0.13	0.004
Tryptophan	90.9	91.6	90.5	91.2	91.4	0.44	0.292
Valine	89.6 ^a^	81.2 ^b^	80.4 ^b^	79.7 ^b^	80.8 ^b^	0.19	0.002
Total EAA	91.0	87.97	87.12	87.07	87.53		
Non-essential amino acids							
Alanine	81.0 ^b^	83.5 ^a^	81.4 ^b^	83.4 ^a^	83.2 ^a^	0.35	0.003
Aspartic Acid	80.5 ^a^	81.5 ^a^	79.5 ^ab^	78.0 ^b^	80.5 ^a^	0.63	0.002
Cysteine	72.8 ^a^	68.1 ^b^	71.6 ^a^	69.8 ^b^	70.0 ^ab^	0.28	0.043
Glutamic Acid	87.1 ^a^	88.5 ^a^	85.5 ^b^	85.8 ^b^	88.0 ^a^	0.23	0.023
Glycine	80.4 ^a^	79.0 ^ab^	78.4 ^b^	78.0 ^b^	77.6 ^b^	0.14	0.012
Proline	83.0	80.0	81.4	81.6	81.8	0.27	0.321
Serine	83.4 ^a^	82.0 ^a^	81.8 ^ab^	80.9 ^b^	79.8 ^b^	0.21	0.020
Tyrosine	93.4 ^a^	83.2 ^b^	83.8 ^b^	83.4 ^b^	83.5 ^b^	0.30	0.032
Total NEAA	82.7	80.725	80.43	80.11	80.55		
Total AA	87.28	84.75	84.14	83.98	84.43		

^a–c^ Means within the same row with different superscripts are significantly different at *p* < 0.05. ^1^ Experimental diets: SBM = reference diet prepared by substituting 25% of the basal diet with soybean meal; SH1 = test diet prepared by substituting 25% of the basal diet with soyhulls sourced from Sadiq Brother Feed, Mandra, Pakistan; SH2 = test diet prepared by substituting 25% of the basal diet with soyhulls sourced from Shabbir Feed, Multan, Pakistan; SH3 = test diet prepared by substituting 25% of the basal diet with soyhulls sourced from Sindh Feeds, Karachi, Pakistan; SH4 = test diet prepared by substituting 25% of the basal diet with soyhulls sourced from Hi-Tech Feeds, Lahore, Pakistan.

**Table 5:** Standardized ileal digestibility (%) of amino acids in laying hens fed experimental diets during phase 2 (32 to 36 weeks of age).

Item	Experimental Diets ^1^					SEM	*p*-Value
	SBM	SH1	SH2	SH3	SH4		
Essential amino acids							
Arginine	93.0 ^a^	87.4 ^b^	85.9 ^c^	87.2 ^b^	87.0 ^b^	0.78	0.002
Histidine	91.5 ^a^	85.8 ^bc^	86.2 ^b^	84.2 ^c^	84.5 ^c^	0.63	0.015
Isoleucine	88.1 ^a^	83.5 ^b^	79.4 ^c^	81.0 ^bc^	83.0 ^b^	0.6	0.031
Leucine	84.2 ^a^	79.6 ^c^	83.2 ^a^	81.0 ^b^	83.0 ^a^	1.01	0.022
Lysine	91.0 ^a^	87.7 ^b^	87.4 ^b^	85.8 ^b^	82.8 ^c^	0.8	0.021
Methionine	89.5	92.5	92	90.2	90	0.64	0.071
Phenylalanine	92.0 ^a^	85.6 ^b^	83.7 ^cd^	84.2 ^c^	82.5 ^d^	0.62	0.003
Threonine	90.0 ^a^	84.4 ^b^	82.0 ^c^	82.2 ^c^	84.2 ^b^	0.66	0.012
Tryptophan	91.3 ^a^	89.5 ^b^	86.0 ^c^	86.4 ^c^	89.0 ^b^	0.77	0.001
Valine	87.4 ^a^	77.6 ^b^	75.2 ^c^	77.4 ^bc^	75.7 ^c^	0.9	0.003
Total EAA	89.8	85.36	84.1	83.96	84.17		
Non-essential amino acids							
Alanine	81.1	80.7	79.4	80.1	79	1.12	0.119
Aspartic Acid	79.3 ^a^	78.5 ^ab^	77 ^b^	76.5 ^b^	77.8 ^b^	1.32	0.004
Cysteine	74.8 ^a^	74.5 ^a^	71.3 ^b^	73.6 ^a^	74.2 ^a^	0.8	0.011
Glutamic Acid	87.2 ^a^	85.7 ^b^	82.2 ^cd^	80.8 ^d^	83.5 ^c^	0.98	0.001
Glycine	78.8	78.3	78	77.5	77.8	1.12	0.224
Proline	82.6	83	82.4	82	81.8	1.2	0.181
Serine	81.0 ^a^	78.3 ^b^	78.9 ^b^	83.4 ^a^	79.4 ^b^	0.86	0.005
Tyrosine	91.0 ^a^	83.6 ^b^	79.4 ^c^	81.5 ^bc^	80.0 ^c^	0.9	0.003
Total NEAA	81.98	80.325	78.575	79.425	79.19		
Total AA	86.32	83.12	81.64	81.94	81.95		

^a–d^ Means within the same row with different superscripts are significantly different at *p* < 0.05. ^1^ Experimental diets: SBM = reference diet prepared by substituting 25% of the basal diet with soybean meal; SH1 = test diet prepared by substituting 25% of the basal diet with soyhulls sourced from Sadiq Brother Feed, Mandra, Pakistan; SH2 = test diet prepared by substituting 25% of the basal diet with soyhulls sourced from Shabbir Feed, Multan, Pakistan; SH3 = test diet prepared by substituting 25% of the basal diet with soyhulls sourced from Sindh Feeds, Karachi, Pakistan; SH4 = test diet prepared by substituting 25% of the basal diet with soyhulls sourced from Hi-Tech Feeds, Lahore, Pakistan.

**Table 6:** Standardized ileal digestibility (%) of amino acids in laying hens fed experimental diets during phase 3 (36 to 40 weeks of age).

Item	Experimental Diets ^1^					SEM	*p*-Value
	SBM	SH1	SH2	SH3	SH4		
Essential amino acids							
Arginine	93.8 ^a^	91.5 ^b^	90.5 ^b^	90.7 ^b^	91.0 ^b^	0.16	0.008
Histidine	90.2 ^a^	85.8 ^bc^	85.5 ^bc^	86.0 ^b^	84.3 ^c^	0.15	0.010
Isoleucine	88.2 ^a^	83.4 ^b^	82.5 ^b^	83.1 ^b^	82.2 ^b^	0.21	0.012
Leucine	84.8	85.0	84.4	85.2	85.5	0.19	0.506
Lysine	91.0 ^a^	89.0 ^b^	87.3 ^c^	88.0 ^bc^	87.3 ^c^	0.26	0.002
Methionine	91.9 ^c^	96.1 ^a^	95.9 ^ab^	94.6 ^b^	95.6 ^ab^	0.11	0.004
Phenylalanine	91.0 ^a^	88.2 ^b^	86.6 ^c^	87.6 ^bc^	86.9 ^c^	0.25	0.005
Threonine	86.4 ^a^	86.2 ^a^	84.2 ^b^	85.0 ^ab^	85.3 ^ab^	0.21	0.023
Tryptophan	88.9 ^b^	91.4 ^a^	89.8 ^ab^	89.8 ^ab^	90.6 ^a^	0.21	0.006
Valine	88.2 ^a^	82.0 ^b^	81.4 ^b^	80.3 ^b^	80.0 ^b^	0.14	0.005
Total EAA	89.44	87.86	86.81	87.03	86.87		
Non-essential amino acids							
Alanine	81.5	83.6	83	82	83.4	0.35	0.062
Aspartic Acid	81.0 ^b^	83.1 ^a^	82.4 ^ab^	81.8 ^b^	79.4 ^c^	0.19	0.005
Cysteine	71.4 ^a^	69.5 ^b^	69.2 ^b^	68.2 ^b^	68.5 ^b^	0.22	0.013
Glutamic Acid	86.8 ^b^	88.3 ^a^	87.8 ^ab^	86.8 ^b^	86.5 ^b^	0.2	0.034
Glycine	81.2 ^a^	78.5 ^b^	77.5 ^b^	77.8 ^b^	77.2 ^b^	0.2	0.002
Proline	81.0	80.0	79.3	78.5	79.0	0.27	0.081
Serine	84.2 ^a^	80.0 ^c^	82.4 ^b^	82.7 ^b^	82.5 ^b^	0.27	0.016
Tyrosine	92.0 ^a^	84.0 ^b^	82.8 ^c^	82.5 ^c^	82.0 ^c^	0.12	0.002
Total NEAA	82.39	80.88	80.55	80.04	79.81		
Total AA	86.31	84.76	84.03	83.92	83.73		

^a–c^ Means within the same row with different superscripts are significantly different at *p* < 0.05. ^1^ Experimental diets: SBM = reference diet prepared by substituting 25% of the basal diet with soybean meal; SH1 = test diet prepared by substituting 25% of the basal diet with soyhulls sourced from Sadiq Brother Feed, Mandra, Pakistan; SH2 = test diet prepared by substituting 25% of the basal diet with soyhulls sourced from Shabbir Feed, Multan, Pakistan; SH3 = test diet prepared by substituting 25% of the basal diet with soyhulls sourced from Sindh Feeds, Karachi, Pakistan; SH4 = test diet prepared by substituting 25% of the basal diet with soyhulls sourced from Hi-Tech Feeds, Lahore, Pakistan.

## Data Availability

The data that support the findings of this study are available from the corresponding author upon reasonable request.
